# P38 Mitogen-Activated Protein Kinase Protects Against Retinoblastoma Through Regulating USP22/SIRT1/SOST Axis

**DOI:** 10.3389/fonc.2022.781247

**Published:** 2022-03-09

**Authors:** Xiaoming Huang, Jianfeng Wan, Fei Liu, Yang Liu, Lina Wang, Sidi Zhao, Tong Wu, Fengyuan Sun

**Affiliations:** ^1^ Department of Orbital Disease and Oculoplastic Surgery, Sichuan Eye Hospital, AIER Eye Hospital Group, Chengdu, China; ^2^ Department of Orbital Disease and Oculoplastic Surgery, Tianjin Medical University Eye Hospital, Tianjin, China; ^3^ Respiratory and Critical Care Medicine Department, Yidu Central Hospital, Weifang, China; ^4^ Research and Development Department, Microsensor Labs, Chicago, IL, United States

**Keywords:** retinoblastoma, p38 MAPK, USP22, SIRT1, SOST

## Abstract

Retinoblastoma (RB) is the most common intraocular malignancy in children. It has been previously reported that p38 MAPK is related to the pathogenesis of RB. Here we aim at investigating how p38 MAPK affected RB progression through mediating USP22/SIRT1/SOST axis. In this study, Thirty-two cases of RB and normal retinal tissues were collected. The expression of p38 MAPK, phosphorylation of p38 MAPK (P-p38 MAPK), USP22, SIRT1 and SOST in clinical tissues and cells was measured using RT-qPCR, IHC assay or western blot analysis. Cell proliferation was detected by CCK-8. Apoptosis rate of cells was examined by flow cytometry. Cell migration was evaluated using scratch test. Cell invasion ability was examined by Transwell assay. Co-immunoprecipitation (CO-IP) was utilized to measure the deubiquitination of USP22 on SIRT1. *In vivo*, mice were respectively injected with plasmids and the tumor growth as well as the tumor weight were detected. Results showed that p38 MAPK, P-p38 MAPK and SOST were poorly expressed in RB tissues and cells whereas USP22 and SIRT1 were overly expressed. P-p38 MAPK inhibited the expression of USP22, and overexpression of USP22 eliminated the inhibitory roles of P-p38 MAPK on tumor growth, as well as cell proliferation, migration and invasion. USP22 stabilized and promoted the expression of SIRT1 through its deubiquitination function. Silencing the expression of SIRT1 contributed to boosted expression of SOST, thus suppressing the growth of tumor cells. Collectively, the phosphorylation of p38 MAPK regulates the SIRT1/SOST axis to protect against RB *via* silencing USP22. The findings present some cues for a further approach to RB.

## Introduction

Retinoblastoma (RB) is the most common primary intraocular malignancy in childhood, but it is not a common pediatric cancer with the incidence of live births constant at 1: 15,000-1: 20,000 globally (1). Moreover, RB is the first tumor to raise concerns about the genetic causes of cancer and has a mortality rate of about 70 percent in low- and middle-income countries where the most affected children live in spite of a thorough and good understanding on its etiology (2). Early diagnosis of RB can save a child’s life and vision, but there is evidence in the community that many children around the world are diagnosed late (3). Currently, intra-arterial and intravitreal chemotherapy have become prospective ways to save the eyes (4). A prior report has revealed that p38 MAPK can inhibit the development of RB through promoting apoptosis (5). P38 MAPK interacts and activates related signaling molecules through phosphorylation, thereby participating in the regulation of a wide range of cellular processes (6). And activation of p38 MAPK pathway exerts significant effects on the apoptosis of Y79 cells (7). Additionally, in Hela cells, p38 MAPK can inhibit the expression ubiquitin-specific protease 22 (USP22) deubiquitinase (8). USP22 is known as a member of the ubiquitin-specific protease, a well-defined protein that promotes poor prognosis, invasion, and metastasis, and is also involved in the maintenance of cancer stem cells (9). Research has found that USP22 accelerates the occurrence of RB (10). Surprisingly, loss-of-function of USP22 promotes the senescence and apoptosis of human RB cells through suppressing the TERT/P53 pathway 10. Furthermore, USP22 can stabilize and promote the expression of sirtuin-1 (SIRT1) through its deubiquitination enzyme function (11). SIRT1 is an NAD (+) -dependent histone deacetylase that functions as critical roles in a variety of biological processes, including lifespan, stress response, and cell survival (12). Likewise, SIRT1 has been reported to promote the development of RB. Relative literature on the relation between SIRT1 and RB has expounded that silencing of the expression of Bcl-2 and SIRT1 in RB can inhibit the proliferation of RB cells and promote their apoptosis (13). SIRT1 directly and negatively regulates Sclerostin (SOST) gene expression by deacetylation of histone 3 at lysine 9 on the SOST promoter (14). SOST is a glycoprotein mainly derived from bone cells and an important regulator of bone remodeling (15). SOST can attenuate the progression of RB and down regulation of SOST promotes the proliferation and invasion of RB cells and reduces their apoptosis by activating the Wnt/he-catenin signaling pathway (16). Therefore, this study aims at investigating how p38 MAPK affected in RB through mediating USP22/SIRT1/SOST axis.

## Materials and Methods

### Ethical Approval

The study protocol was approved by the Ethics Committee of Tianjin Medical University Eye Hospital and conducted in line with the *Declaration of Helsinki*. The parents or guardians of all participants provided written informed consent and agreed to use the tissues for a comprehensive RB trial. Experiments involving animals were performed in accordance with the principles of experimental animals of the National Institutes of Health.

### Clinical Samples

Patients who were hospitalized in Tianjin Medical University Eye Hospital from January 2018 to March 2019 were selected as the study subjects. All the participants were pathologically diagnosed as RB and received no radiotherapy or chemotherapy with neither dermal blastoma or extraocular metastasis, nor neovascular glaucoma, or refractive opacity. Moreover, they did not suffer dysfunction of liver and kidney or hearing impairment. A total of 32 RB tissues were collected and adjacent normal tissues were used as control. All tissue samples were rapidly frozen in liquid nitrogen after nucleation for later use.

### Cell Culture and Transfection

Human normal retinal epithelial cell line ARPE-19 and RB cell lines (SO-Rb50 and HXO-RB44) purchased from cell bank of China Center for Type Culture Collection were cultured with dulbecco’s modified Eagle’s medium (DMEM) (Gibco Life Technologies, Inc., Grand Island, NY, USA). RB cell line Y79 cells (obtained from St. JudeChildren’s Research Hospital) were cultured with RPMI-1640 medium (Gibco Life Technologies). The cells were treated with 0.25% pancreatin and passaged at 1: 3. When the degree of cell growth and confluence reached 80% - 90% (logarithmic growth phase), the cells were taken for subsequent experiments. To investigate p38 MAPK signal functions, Y79 RB cells (4 × 10^5^ cells/well) were inoculated in a 6-well plate with stable plasmids oe- p38 MAPK and treated with p38 MAPK phosphorylation activator MKK6 (ab95252, Abcam, Cambridge, UK), cells also transfected with stable plasmids oe-USP22, sh-USP22, sh-SIRT1, oe-SIRT1, sh-SOST or their NC individually or together. Transfection sequences and plasmids were purchased from Shanghai GenePharma Co., Ltd. (Shanghai, China).

### Immunohistochemistry (IHC)

IHC assay was used to detect the expression of p38 MAPK and USP22 in clinical samples. The obtained 5 μm paraffin-embedded tissues were dewaxed with xylene and hydrated with a series of gradient alcohols. Then the sections were washed with distilled water and incubated in citrate buffer. Hydrogen peroxide (0.3%) was used to block endogenous peroxidase activity. Sections were blocked with 1% bovine serum albumin (BSA) and 20% mouse serum (M5905, Sigma-Aldrich, St. Louis, MO, USA) in phosphate buffered saline (PBS) for 10 min. Then the sections were incubated with rabbit anti-p38 MAPK (8690S; 1: 400, Cell Signaling Technology, Danvers, MA, USA), Phospho-p38 MAPK (44-684G; 1:100, Cell Signaling Technology, Danvers, MA, USA),USP22 (ab217968; 1: 100, Abcam, Cambridge, UK) and Ki67 (ab92742; 1:1000, Abcam) at 4°C overnight. Afterwards, the sections were incubated with horseradish peroxidase (HRP)-conjugated goat anti-rabbit secondary antibody (ab6702; 1: 2000, Abcam) for 1 h, added with horseradish peroxidase-labeled streptavidin protein working solution (0343-10000U, Yimo Biotechnology Co., Ltd., Beijing, China), placed at 37°C for 20 min. And 2, 4-diaminobutyric acid (DAB) (ST033, Whiga Technology Co., Ltd., Guangzhou, China) was used for color development, and the sections were washed after color development. Hematoxylin (PT001; Shanghai Bogoo Biotechnology. Co., Ltd, Shanghai, China) was used to counterstain for 1 min and then washed. Afterwards, the sections were returned to blue with 1% ammonia water dehydrated with gradient of alcohol, and cleared with xylene and sealed. The sections were observed and photographed under a microscope. The quantification of the IHC images was performed using Image-J software (NIH, Bethesda, MD).

### RT-qPCR

Total RNA was extracted using Trizol reagent (15596026; Invitrogen Inc.), and reversely transcribed to cDNA according to the PrimeScript RT reagent Kit (RR047A, Takara, Japan). Fast SYBR Green PCR kit (Applied biosystems) and ABI PRISM 7300 RT-PCR system (Applied biosystems) were used for RT-qPCR detection of the synthesized cDNA, and 3 parallel wells were set. GADPH was used as an internal reference to analyze the relative expression of the expressed genes of p38 MAPK, USP22, SIRT1 and SOST by 2^-ΔΔCt. △△Ct^ = (average Ct value of target gene in experimental group - average Ct value of housekeeping gene in experimental group) - (average Ct value of target gene in control group - average Ct value of housekeeping gene in control group). The primer sequences were shown in **Supplementary Table 1**.

### Western Blot

RB tissues and normal retinal tissues or cells were lysed to isolate total protein. Proteins were separated by 10% sodium dodecyl sulfide-polyacrylamide gel electrophoresis (SDS-PAGE), and then electrotransferred to nitrocellulose membrane (Millipore, Temecula, CA, USA) and blocked with 5% BSA at room temperature for 2 h to block nonspecific binding. Primary rabbit anti-p38 MAPK, anti-P-p38 MAPK, USP22, SIRT1, SOST, and GAPDH (ab128915; 1: 10000, Abcam) were incubated with the membrane overnight at 4°C. The membrane was then incubated with HRP-labeled goat anti-rabbit secondary antibody (ab6702; 1:2000, Abcam) for 1 h at room temperature, developed using ECL working solution (EMD Millipore, USA) and exposed. Image J analysis software was used to quantify the gray scale of each band in the Western blot Image. GAPDH was taken as internal reference.

### Cell Counting Kit-8 (CCK-8) Assay

Cells were treated with 0.25% trypsin to make a single-cell suspension. After counting, the cells were inoculated at a density of 3-6 × 10^3^ cells/well in a 96-well plate with a volume of 200 μL/well and 6 parallel wells were set for incubation. Then culture plates were removed respectively at 24 h, 48 h, and 72 h. Each well was added with 10 μL CCK8 (VP757; Dojindo Co., Ltd., Kumamoto, Japan) for another 2 h incubation. The optical density (OD) values of each well were read at 450 nm with the enzyme-linked immunoassay (biobase-el10a, Jinan Boxin biotechnology Co., Ltd., Jinan, China). The cell activity curve was drawn with the time point as the abscissa and OD value as the ordinate.

### Transwell Assay

The Matrigel was spread on the upper Transwell chamber in each well and incubated at 37°C for 2 h. The fibronectin was coated on the other side of the membrane, and about 200 mL (5 × 10^5^ cells/mL) was added into the upper chamber and incubated for 24 h. Then, the cells on the membrane were wiped away and the membrane was taken away. After being fixed in 4% paraformaldehyde at room temperature for 30 min, hematoxylin staining was performed, followed by PBS washing and ethanol dehydration. The cells penetrating the matrigel were recorded.

### Scratch Test

The cells in the logarithmic phase were digested, counted, and seeded into a six-well plate with 5 × 10^5^ cells/mL cells in each well. With the addition of a medium containing 10% FBS, the cells were cultured in a 5% CO_2_ incubator at 37°C for 24 h. The monolayer cell was scratched by a 10 μL Tip when the cover degree approached 100% with the medium aspirated. The cells were rinsed by PBS and continually incubated in FBS-free medium. The cells were observed under a microscope and photos were taken. After 24 and 48 h of incubation at 37°C in a 5% CO_2_ incubator, the cell migration was observed and photos were taken. The Cell Profiler software was adopted for measuring of the scratch area.

### Flow Cytometry

Annexin V-Fluorescein-5-isothiocyanate (FITC)/propidium iodide (PI) double staining kit (556547; Shanghai Shuojia Biotechnology Co., Ltd., Shanghai, China) was used to detect the apoptosis of RB cells in each group. Firstly, 10×Binding Buffer was diluted with deionized water and the cells in each group were centrifuged at room temperature at 2000 rpm for 5 min. The cells were resuspended with 1×PBS, followed by centrifugation at 300×g for 5-10 min. After that, 300 μL1¡ÁBinding Buffer was added to suspend cells. After 5 μL Annexin V-FITC was added and mixed, the cells were incubated for 15 min in a dark chamber. And 5 μL of PI was added for cell apoptosis detection using flow cytometer (FACSVerse/Calibur/AriaIISORP, BD, USA). The excitation wavelength was AT 480 nm, FITC was detected at 530 nm and PI was detected at greater than 575 nm.

### Co-Immunoprecipitation (CO-IP)

The transfected Y79 cells were cultured for 42 h and treated with 10 μM MG132 (m7449-1ML, Sigma-Aldrich) for 6 h. Then the cells were lysed with the lysis buffer (50 mM Tris - HCl (pH 7.4), 150 mM NaCl, 10% glycerin, 1 mM EDTA, 0.5% NP-40 and protease inhibitor mixture). The cell fragments were removed by centrifugation and the supernatant was taken. The supernatant was incubated with anti-HA or anti-Flag antibody (F2555-100UL, 1:250, Sigma-Aldrich) and Protein G Magnetic Beads (9006S, Santa Cruz Biotechnology) for 2 h. The beads were boiled at 100°C for 5 min after the lysate was washed for 3 times. The proteins were separated by SDS-PAGE, transferred to cellulose nitrate film (Millipore, Temecula, CA, USA), and imprinted.

### Animal Studies

Forty-five BALB/c nude mice weighing 10 - 12 g (male, 3 - 5 weeks) were purchased from Beijing Vital River Laboratory Animal Technology Co., Ltd. (Beijing, China). All the mice met the standard of Ministry of Public Health of China for laboratory animals with no specific pathogen (SPF). The mice were divided into three groups (n = 15). The mice were injected with plasmids expressing oe-NC + sh-NC, oe-P-p38 MAPK + sh-NC or oe-P-p38 MAPK + sh-SOST. The concentration of cell suspension of Y79 cells was adjusted to 1 × 10^6^ cells/mL with PBS solution, and 50 μL cell suspension was subcutaneous injected into the right side of each mouse. After 35 days, the mice were anesthetized with pentobarbital sodium (100 mg/kg, P3761; Sigma-Aldrich), the tumors were dissected and isolated, and the short (a) and long (b) diameters of the tumors were recorded with a vernier caliper. The tumor volume was calculated according to the formula V = (a^2^ × b)/2, and the tumor mass was weighed with a balance. The measurement was repeated 3 times for each group.

### Statistical Analysis

Statistical analysis was conducted by SPSS 21.0 (IBM, Armonk, New York, USA). Measurement data were expressed by mean ± standard deviation. Unpaired *t* test was performed for comparisons of data with normal distribution and homogeneous variance between two groups. One-way analysis of variance (ANOVA) was conducted for multiple group comparison, followed by Tukey’s *post hoc* test. Statistical significance was set up at *p* < 0.05.

## Results

### P38 MAPK and P-p38 MAPK Is Poorly Expressed in RB Tissues and Cells

**Figure 1:**
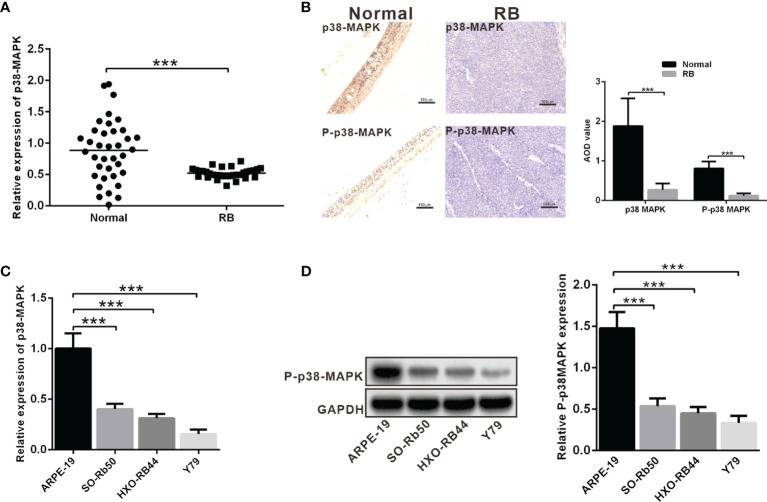
The expression of p38 MAPK and P-p38 MAPK is depleted in RB tissues and cells. **(A)** p38 MAPK expression in RB tissues was detected using RT-qPCR (n = 32). **(B)** Expression of p38 MAPK and P-p38 MAPK in clinical samples was measured using IHC assay (n = 32). The p38 MAPK and P-p38 MAPK average optical density (AOD, analysis by soft wear Image J) in RB were significant lower than in normal retina tissues. **(C)** p38 MAPK expression in ARPE-19, SO-Rb50, HXO-RB44 and Y79 cells was assessed by RT-qPCR. **(D)** P-p38 MAPK expression in ARPE-19, SO-Rb50, and HXO-RB44 and Y79 cells was assessed by western blot analysis. ***p < 0.001. The measurement data was expressed by mean ± standard deviation. The two groups of data conforming to the normal distribution were compared using unpaired *t* test.

It has been reported that the kinase MAPK can inhibit the occurrence of RB by activating apoptosis. To investigate the role of *p38 MAPK* in RB, the expression of *p38 MAPK* in normal retinal tissues and RB tissues was detected using RT-qPCR, which showed that *p38 MAPK* expression was significantly lower in RB tissues in comparison with that in normal retinal tissues (**Figure 1A**). Meanwhile, the observation of IHC assay displayed that and phosphorylation of p38 MAPK (P-p38 MAPK) expression was remarkably decreased in RB tissues (**Figure 1B**). Moreover, p38 MAPK expression in human normal retinal epithelial cell line ARPE-19 and RB cell lines (SO-Rb50, HXO-RB44 and Y79) was also measured using RT-qPCR and western blot analysis, results of which exhibited that the expression of p38 MAPK and P-p38 MAPK in RB cell lines was significantly lower than that in normal retinal cells, and p38 MAPK and P-p38 MAPK expression was the lowest in Y79 cells (**Figures 1C, D**). Hence, Y79 cells were selected for subsequent experiments.

### Overexpression of P-p38 MAPK Inhibit Proliferation, Migration, and Invasion and Promote Apoptosis of Y79 Cells

**Figure 2:**
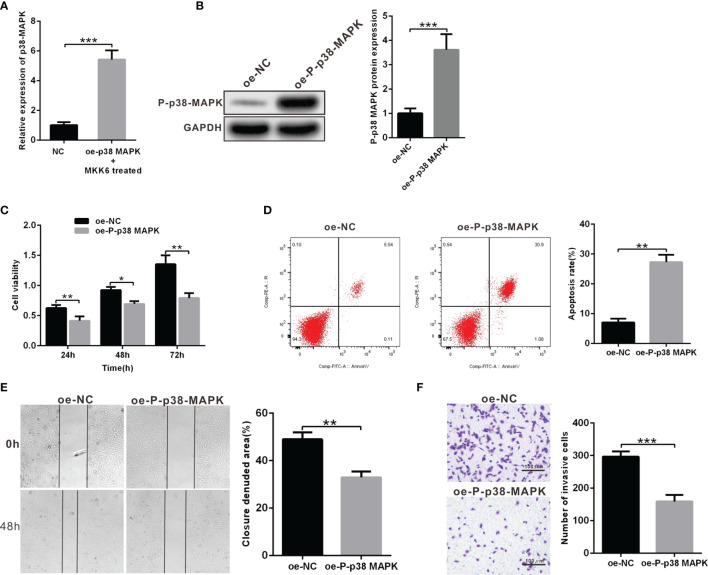
Overexpression of P-p38 MAPK contributes to inhibited tumor cell proliferation, metastasis and invasion and accelerated apoptosis. **(A)** p38 MAPK expression in oe-p38 MAPK or NC-transfected Y79 cells was measured using RT-qPCR. **(B)** P-p38 MAPK expression in Y79 cells was measured using western blot analysis. **(C)** Cell viability was determined using CCK-8. **(D)** Cell apoptosis was detected using flow cytometry. **(E)** Cell migration was measured by scratch test. **(F)** Cell invasion was tested using Transwell assay. *p < 0.05 vs. oe-NC-transfected Y79 cells. **p < 0.01, ***p < 0.001. The measurement data was expressed by mean ± standard deviation. The two groups of data conforming to the normal distribution were compared using unpaired *t* test.

In order to further explore the role of p38 MAPK in RB tumorigenesis and development, we used Y79 cells to construct the model of overexpression. oe-p38 MAPK-transfected Y79 cells contributed to highly increased mRNA expression of p38 MAPK and treated with p38 MAPK phosphorylation activator MKK6 to elevated P-p38 MAPK protein expression, which demonstrated the success of overexpression model (**Figures 2A, B**). CCK-8 was utilized to detect Y79 cell viability, which showed that cell viability in the Y79 cells transfected with oe-P-p38 MAPK was notably decreased than that in the oe-NC-transfected Y79 cells (**Figure 2C**). Simultaneously, cell apoptosis in the Y79 cells transfected with oe-P-p38 MAPK was dramatically increased compared with that in the oe-NC-transfected Y79 cells (**Figure 2D**). In terms of migration and invasion, we found that oe-P-p38 MAPK-transfected Y79 cells showed significantly decreased capacity in migration and invasion when compared to that in the oe-NC-transfected Y79 cells (**Figures 2E, F**). Collectively, highly expressed P-p38 MAPK promoted cell apoptosis and impaired the abilities of invasion and migration.

### Overexpression of USP22 Reverses the Inhibitory Role of P-p38 MAPK Overexpression in Tumor Cells

**Figure 3:**
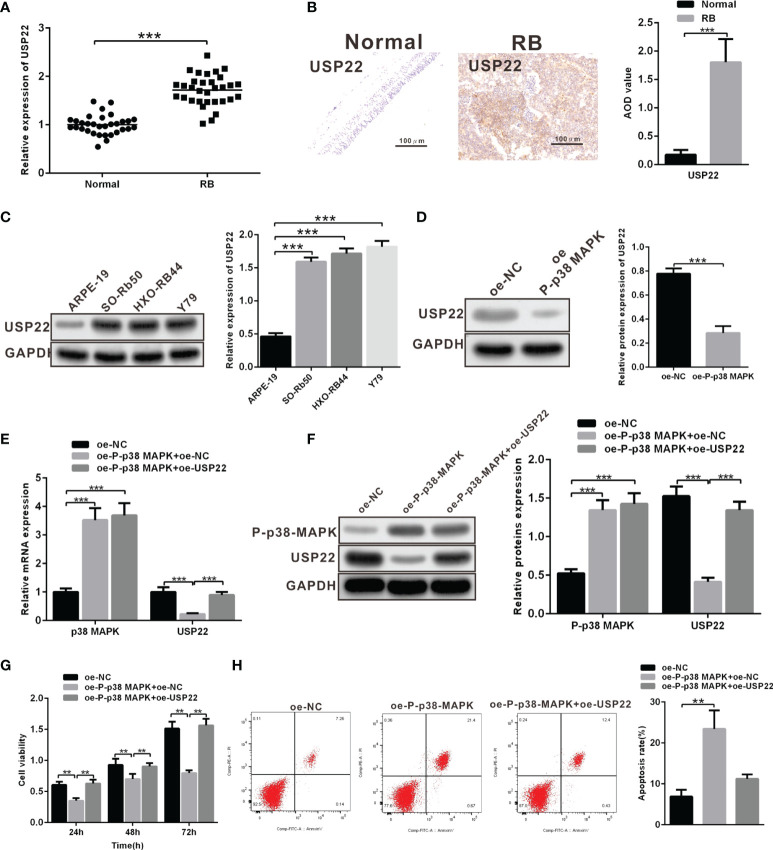
Overexpression of USP22 reverses the inhibitory roles of P-p38 MAPK on RB cell functions. **(A)** USP22 expression in clinical samples was detected using RT-qPCR (n = 32). **(B)** USP22 expression in clinical samples was detected using IHC assay. **(C)** USP22 expression in RB cell lines was measured using western blot analysis. **(D)** USP22 expression in Y79 cells that over expressed P-p38 MAPK was detected by western blot analysis. **(E, F)** The expression of P-p38 MAPK and USP22 after overexpression of P-p38 MAPK and USP22 was respectively determined by the means of RT-qPCR and western blot analysis. **(G)** Cell viability was detected using CCK-8. **(H)** Cell apoptosis was measured using flow cytometry. **p < 0.01, ***p < 0.001. The measurement data was expressed by mean ± standard deviation. The two groups of data conforming to the normal distribution were compared using unpaired *t* test.

It has been reported that in Hela cells, p38 MAPK can inhibit the expression of deubiquitination enzyme USP22. In addition, USP22 can promote the development of RB. These results suggest that p38 MAPK may inhibit the development of RB by regulating USP22. We first examined the expression of USP22 in clinical samples and three RB cell lines, results of which displayed that the mRNA and protein levels of USP22 in tumor tissues were significantly higher than those in the normal retinal cells (**Figures 3A, B**). And the expression of USP22 in RB cells was also higher than that of normal retinal epithelial cells (**Figure 3C**). These results suggested that USP22 was highly expressed in RB. Further, we investigated whether p38 MAPK could regulate the expression of USP22 in RB. We found that USP22 expression in oe-P-p38 MAPK-transfected Y79 cells was significantly lower than that of oe-NC-transfected Y79 cells (**Figure 3D**). To further confirm whether P-p38 MAPK played a role by inhibiting the expression of USP22, Y79 cells were co-transfected with oe-P-p38 MAPK and oe-USP22, and the results claimed that cells co-transfected with oe-P-p38 MAPK and oe-USP22 reversed the reduction of USP22 expression caused by oe-P-p38 MAPK (**Figures 3E, F**). In the meantime, the results of CCK-8 and flow cytometry also exhibited that decreased cell proliferation and increased apoptosis due to overexpression of p38 MAPK could be reversed by overexpression of USP22 (**Figures 3G, H**). The above results showed that P-p38 MAPK could inhibit the expression of USP22, thereby inhibiting the proliferation, migration and invasion and promoting apoptosis in RB cells, while overexpression of USP22 could reverse the effect of P-p38 MAPK on RB cells.

### USP22 Stabilizes SIRT1 Expression Through Deubiqtination to Inhibit SOST Expression

**Figure 4:**
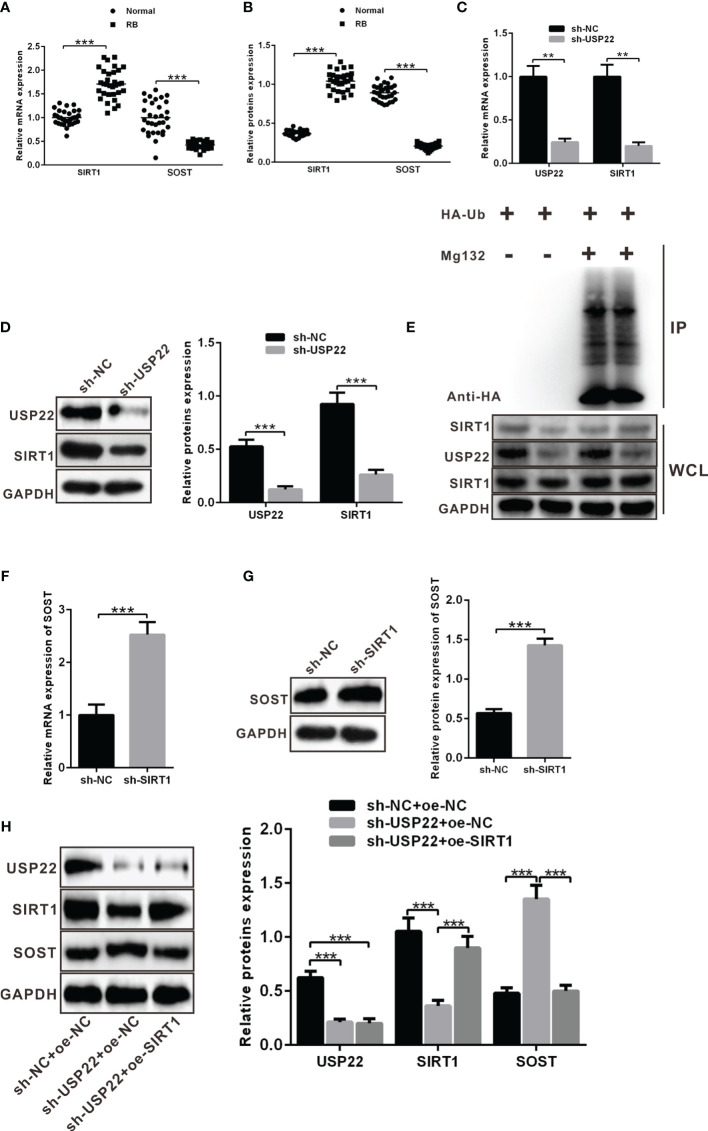
USP22 positively regulates SIRT1 expression through deubiquitination to suppress the expression of SOST. **(A, B)** SIRT1 and SOST expression in clinical samples was measured using RT-qPCR and western blot analysis (n = 32). **(C, D)** SIRT1 expression was detected when USP22 expression was knockdown using RT-qPCR and western blot analysis. **(E)** Deubiquitination of SIRT1 by USP22 was verified in Y79 cells and WCL was an abbreviation of whole cell lysates. **(F, G)** SOST expression was detected when SIRT1 expression was silenced using RT-qPCR and western blot analysis. **(H)** The expression of SOST was regulated by SIRT1 and USP22 detected by western blot analysis. **p < 0.01, ***p < 0.001. The measurement data was expressed by mean ± standard deviation. The two groups of data conforming to the normal distribution were compared using unpaired *t* test.

A prior report has noted that USP22 can stabilize and promote the expression of SIRT1 *via* its function of deubiquitination enzyme. Moreover, SIRT1 can promote the development of RB. SIRT1 directly and negatively regulates SOST gene expression by deacetylating histone 3 at lysine 9 at the SOST promoter. SOST can inhibit the development of RB. We wondered whether USP22 affected the development of RB by acting on SIRT1. Similarly, SIRT1 and SOST expression in RB tissues was detected using RT-qPCR and western blot analysis, which displayed that SIRT1 was highly expressed in RB tissues while SOST was poorly expressed (**Figures 4A, B**). In order to further explore the relationship between USP22 and SIRT1, Y79 cells were treated with plasmids expressing sh-NC and sh-USP22. And detection of SIRT1 expression showed that SIRT1 expression was downregulated in sh-USP22-transfected Y79 cells (**Figures 4C, D**). Post 42 h of co-transfection, the transfected Y79 cells were treated with 10 μM of MG132 for 6 h. Deubiquitination of SIRT1 protein was detected so as to validate the effect of USP22 on SIRT1 through deubiquitination of SIRT1 and we observed that the level of ubiquitination was increased in sh-USP22-transfected cells and SIRT1 expression was significantly decreased. And this phenomenon could be effectively reversed by the proteasome inhibitor MG132, suggesting that the effect of USP22 on SIRT1 protein level was mainly regulated by the proteasome pathway. These results claimed that USP22 could deubiquitinate SIRT1, inhibit its ubiquitination and proteasome degradation of SIRT1, and thus enhance the expression of SIRT1 protein (**Figure 4E**). Subsequently, Y79 cells were co-transfected with sh-NC and sh-SIRT1 to verify whether SIRT1 inhibited the expression of SOST. And the results showed that the expression of SOST in sh-SIRT1-transfected Y79 cells was strikingly elevated in comparison with that in sh-NC-transfected Y79 cells (**Figures 4F, G**). SOST expression was decreased in the Y79 cells while the expression of SOST was boosted with the addition of sh-SIRT1. This demonstrated that SIRT1 could curb the expression of SOST. Therefore, the expression of SOST was increased when SIRT1 expression was inhibited. Next, we aimed to investigate the interaction among USP22, SIRT1 and SOST. USP22 and SIRT1 expression was upregulated in Y79 cells when compared to that in normal retina cells. However, transfection of sh-USP22 in Y79 cells contributed to downregulated expression of USP22 and SIRT1 but elevated SOST expression. Moreover, co-transfection of sh-USP22 and oe-SIRT1 in Y79 cells resulted in decreased expression of USP22 but increased SIRT1 expression, which ultimately led to the reduction on SOST expression (**Figure 4H**). Taken all, the high expression of USP22 in RB promoted the expression of SIRT1 and inhibited the expression of SOST.

### USP22 Accelerates the Proliferation, Migration, Invasion of RB Cells and Inhibits Cell Apoptosis Through SIRT1/SOST Axis

**Figure 5:**
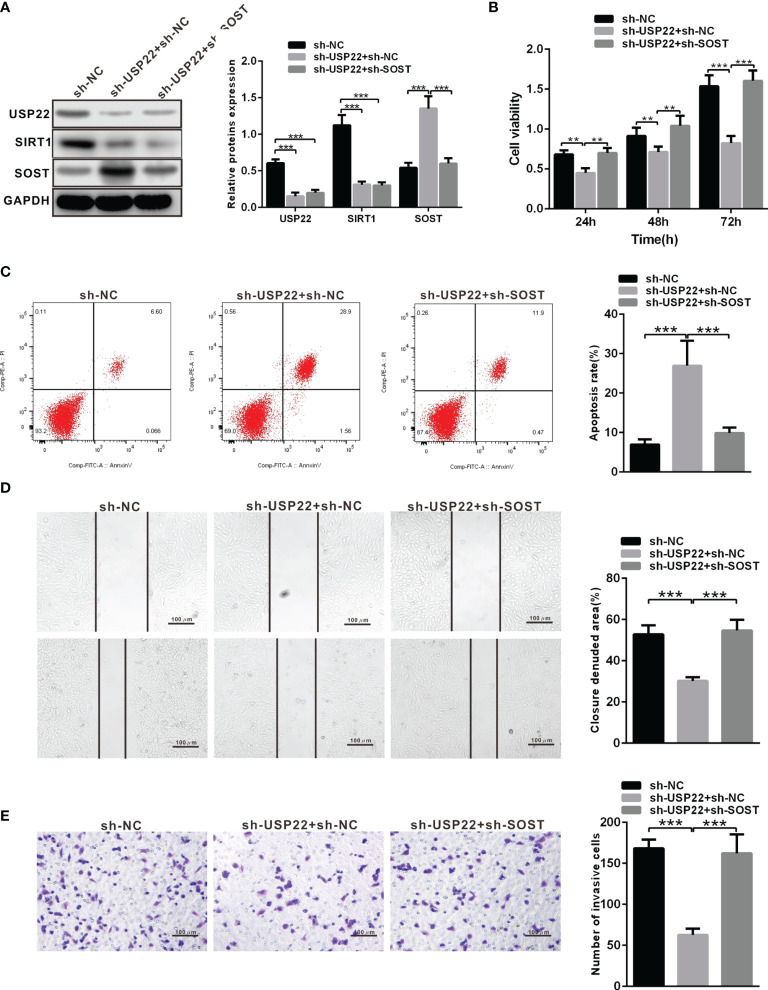
USP22 promotes the occurrence of RB through SIRT1/SOST axis. **(A)** USP22, SIRT1 and SOST expression in sh-USP22-transfected Y79 cells was detected using western blot analysis. **(B)** Cell viability was measured by CCK-8. **(C)** Cell apoptosis was measured using flow cytometry. **(D)** Cell migration was detected using scratch test. **(E)** Cell invasion was assessed by Transwell assay. **p < 0.01, ***p < 0.001. The measurement data was expressed by mean ± standard deviation. The two groups of data conforming to the normal distribution were compared using unpaired *t* test.

According to above results, USP22 stabilized the protein expression of SIRT1 to inhibit SOST expression. Now we silenced the expression of SP22 and SOST in Y79 cells. The protein expression of USP22 and SIRT1 was restrained while SOST expression was raised in sh-USP22-transfected Y79 cells (**Figure 5A**). And the proliferation, metastasis and invasion ability were also weakened but apoptosis was enhanced after Y79 cells were transfected with sh-USP22 (**Figures 5B–E**). Co-transfection of sh-USP22 and sh-SOST not only contributed to downregulated protein expression of USP22 and SIRT1 but also decreased SOST expression (**Figure 5A**). And cell proliferation, migration and invasion ability were promoted whereas the apoptosis was inhibited brought by co-transfection of sh-USP22 and sh-SOST in Y79 cells (**Figures 5B–E**). That was, co-transfection of sh-USP22 and sh-SOST could reverse the biological effect of transfection of sh-USP22 alone. Above all, USP22 accelerated the proliferation, migration and invasion of RB cells and inhibited cell apoptosis through SIRT1/SOST axis.

### P-p38 MAPK inhibits the Development of RB Through USP22/SIRT1/SOST AXIS

**Figure 6:**
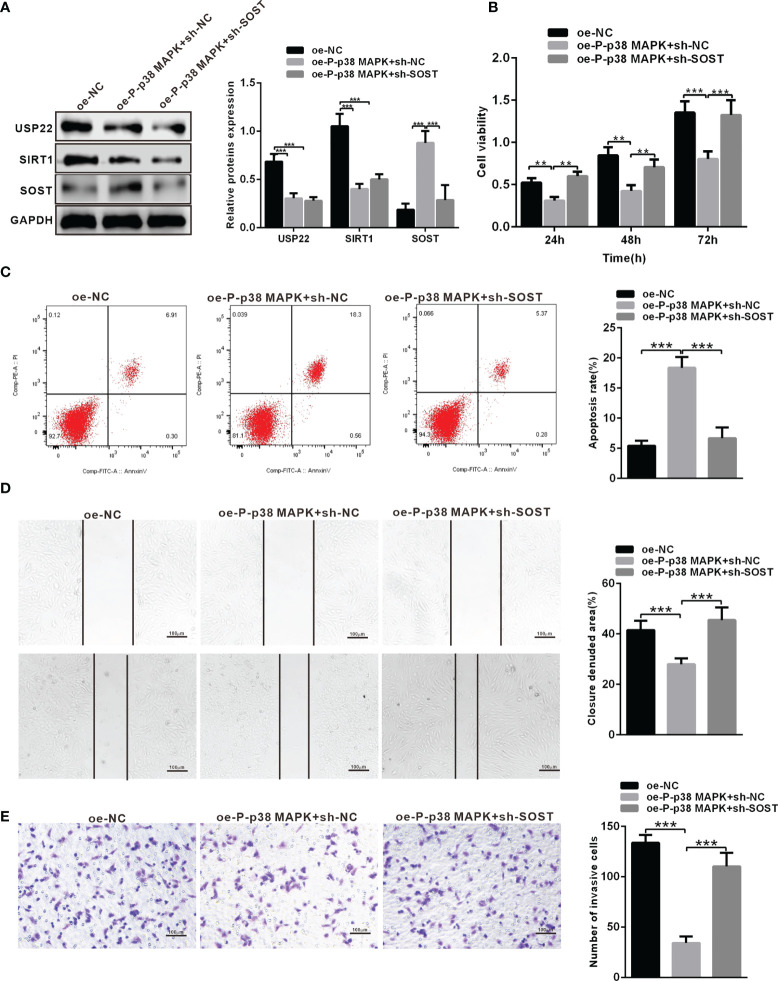
P-p38 MAPK inhibits the development of RB by USP22/SIRT1/SOST axis. **(A)** The expression of USP22, SIRT1 and SOST in the transfected cells was examined by western blot analysis. **(B)** Cell activity was detected using CCK-8. **(C)** Cell apoptosis was tested using flow cytometry. **(D)** Cell migration was detected using scratch test. **(E)** Cell invasion was measured using Transwell assay. **p < 0.01, ***p < 0.001. The measurement data was expressed by mean ± standard deviation. The two groups of data conforming to the normal distribution were compared using unpaired *t* test.

To investigate the effect of p38 MAPK on the development of RB *in vitro.* Y79 cells were transduced with oe-NC + sh-NC, oe-P-p38 MAPK + sh-NC or oe-P-p38 MAPK + sh-SOST. Western blot analysis was performed to determine the expression of P-p38 MAPK, USP22, SIRT1 and SOST, which exhibited that P-p38 MAPK and SOST expression was restored while USP22 and SIRT1 expression was reduced in Y79 cells co-transfected with oe-P-p38 MAPK + sh-NC in comparison with that oe-NC + sh-NC co-transfected Y79 cells (**Figure 6A**). P-p38 MAPK expression was elevated whereas USP22, SIRT1 and SOST expression was all inhibited in Y79 cells co-transfected with oe-P-p38 MAPK + sh-SOST when compared to that oe-NC + sh-NC co-transfected Y79 cells. Cells co-transfected with oe-P-p38 MAPK + sh-NC showed reduced proliferation, migration and invasion ability but increased apoptosis in Y79 cells, which was opposite in cells treated with oe-P-p38 MAPK+ sh-NC compared with that in cells co-transfected oe-NC + sh-NC (**Figures 6B–E**).

### P-p38 MAPK Inhibits the Development of RB *In Vivo*

**Figure 7:**
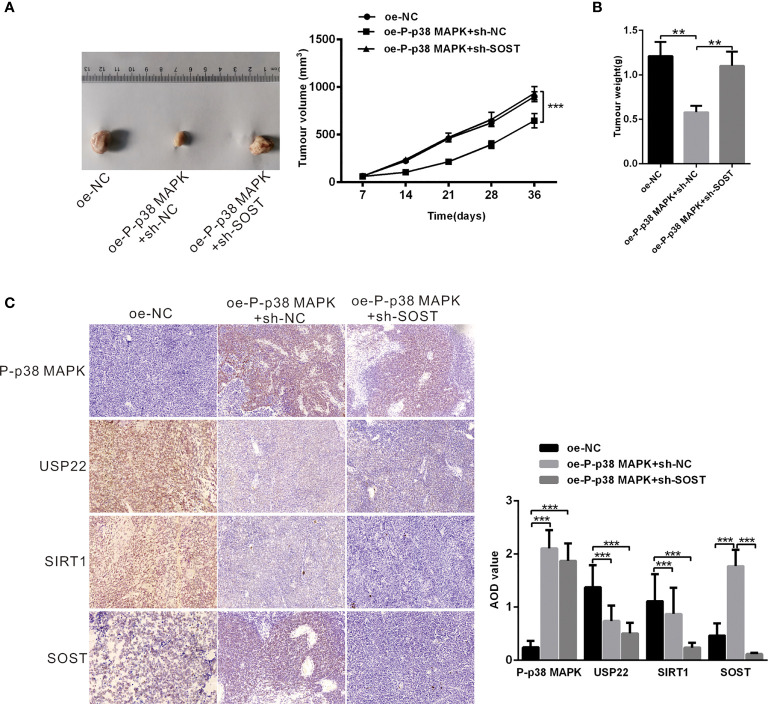
P-p38 MAPK inhibits the development of RB by USP22/SIRT1/SOST axis *in vivo*. **(A)** tumor formation in mice injected with plasmids expressing oe-NC + sh-NC, oe-P-p38 MAPK + sh-NC or oe-P-p38 MAPK + sh-SOST was observed (n = 15). Tumor growth curve was drawn. **(B)** Tumor weight was recorded. **(C)** P-p38 MAPK, USP22, SIRT1 and SOST expression in tumor tissues was measured using IHC assay. **p < 0.001, ***p < 0.001. The measurement data was expressed by mean ± standard deviation. The two groups of data conforming to the normal distribution were compared using unpaired *t* test.

Mice were respectively injected with plasmids expressing oe-NC + sh-NC, oe-P-p38 MAPK + sh-NC or oe-P-p38 MAPK + sh-SOST into subcutaneous adipose tissues to form tumor. The tumor growth curve and weight of mice were measured and the results exhibited that tumor growth and weight in mice infected with oe-P-p38 MAPK + sh-NC were reduced in comparison with that in mice infected with oe-NC + sh-NC or oe-P-p38 MAPK + sh-SOST (**Figures 7A, B**). Additionally, tumor tissues in nude mice were observed by IHC staining, and the results found that USP22 and SIRT1expression in mice injected with plasmids expressing oe-P-p38 MAPK + sh-NC and oe-P-p38 MAPK + sh-SOST were lower than that in mice expressing oe-NC + sh-NC SOST expression in mice injected with plasmids expressing oe-P-p38 MAPK + sh-NC was lower than that in mice expressing oe-NC + sh-NC and oe-P-p38 MAPK + sh-SOST(**Figure 7C**). Taken all together, overexpression of P-p38 MAPK inhibited the growth of RB in nude mice may through affected USP22/SIRT1/SOST axis.

## Discussion

**Figure 8:**
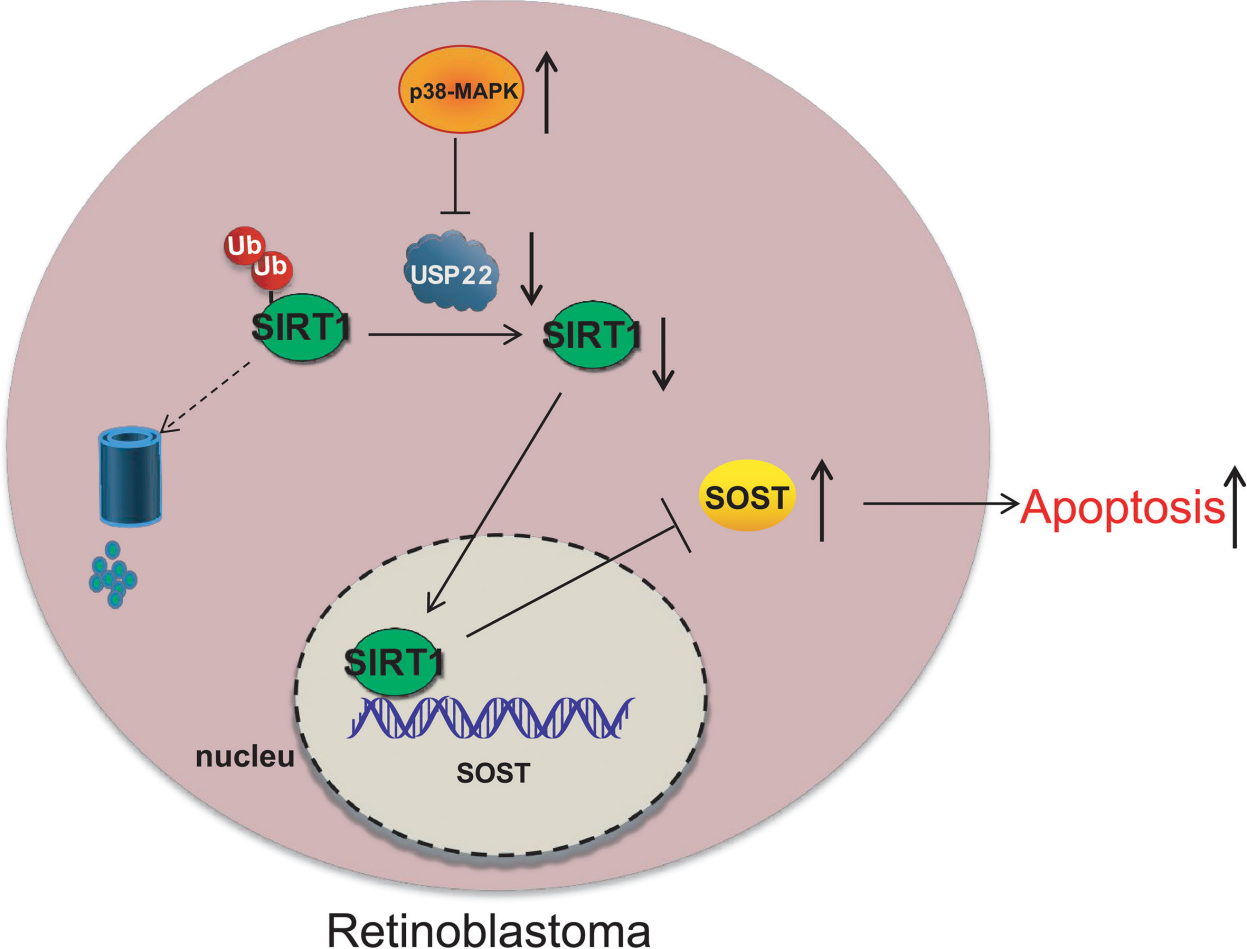
p38 MAPK inhibits the occurrence of RB through regulating USP22 expression *via* SIRT1/SOST axis.

RB is a childhood retinal tumor, caused by the inactivation and loss of RB1 protein (17). RB is a rare form of retinal cancer occurred in infants and every year nearly 8,000 children are diagnosed with this horrible disease (4). About 40% of RB are hereditary and are caused by germline mutations in the gene (18). The goal of RB treatment is to save the eyes, maintain vision, and reduce short-term and long-term side effects without risking death due to tumor spread (19). The severity of the disease determines the odds of survival and vision retention of patients (2). Therapeutic methods such as chemotherapy and radiotherapy are quite effective in the treatment of such cancers, but now there is no way to avoid the high mortality induced by the secondary malignancy which is quite common in these patients (20). It has been reported that p38 MAPK can inhibit the development of RB through activating apoptosis (5). Therefore, the objective of this study was to explore the potential mechanism of p38 MAPK in the progression of RB through regulating USP22/SIRT1/SOST axis. Eventually, this study revealed that activation of p38 MAPK decreased the expression of USP22 and SIRT1 to elevate the expression of SOST, thus inhibiting the development of RB. At the beginning of the experiments, we found that p38 MAPK was poorly expressed in the RB tissues and cells. Overexpression of p38 MAPK resulted in inhibited proliferation, migration, and invasion and accelerated apoptosis of tumor cells. Consistent with our findings, Curcumin plays an anti-tumor role in RB cells through the activation of JNK and p38 MAPK pathways, and from this p38 MAPK is inferred to be downregulated in RB cells (7). Quercetin induces apoptosis of Y79 cells by activating JNK and p38 MAPK pathways, while inactivation of JNK and p38 MAPK suppresses Quercetin-mediated caspase-3/-9 activation and inhibits the apoptosis of cancerous Y79 cells (5). And 2-methoxyestradiol induces Bax phosphorylation and apoptosis in human RB cells by activation of p38 MAPK (21). Moreover, acute knockdown of ΔNp63α significantly increases p38MAPK phosphorylation, thus bringing along increased p21 expression and reduced RB protein phosphorylation (22). Subsequently, further experiments displayed that the inhibited effects of overexpression of p38 MAPK on RB cells would be reversed by the addition of oe-USP22. Results of experiments showed that mRNA and protein expression of USP22 was elevated in RB cells, which resulted in enhanced proliferation, migration, and invasion but decreased apoptosis of Y79 cells. Similarly, our results can be supported and confirmed by previously relative report, for example, overexpression of USP22 can significantly enhance cell proliferation ability and telomerase activity, increase TERT expression level, inhibit p53 expression and cell senescence, and reduce apoptosis or DNA damage, and biological behavior of which can be reversed by the silencing of USP22 expression (10). In pharyngeal squamous cell carcinoma, USP22 expression is up-regulated while knockdown of USP22 expression increases the expression level of cyclin P21 and P27, but reduced the level of phosphorylated RB protein, thereby inhibiting the growth and proliferation of Fa Du cells (23). Then, we revealed that the deubiquitinating enzyme USP22 stabilized and promoted the expression of SIRT1 through its deubiquitination function to suppress the expression of SOST. As report goes, USP22 directly interacts with SIRT1 and positively regulates SIRT1 protein expression (24). Moreover, SIRT1 directly and negatively regulates SOST gene expression by deacetylation of histone 3 at lysine 9 on the SOST promoter (14). Accordingly, USP22 promoted the proliferation, migration and invasion of RB cells and inhibited apoptosis through upregulating SIRT1 expression and reducing the expression of SOST. We also found that SOST expression was downregulated in RB cells. Consistent with our results, the mRNA expression of SOST in RB cells is lower than that in normal retina tissues, and depletion of SOST promotes the proliferation and invasion of RB cells and reduces tumor cells apoptosis (16). Finally, we verified *in vivo* that p38 MAPK inhibited the occurrence of RB through the regulating USP22/SIRT1/SOST axis. However, as little evidence has shown the interaction among p38 MAPK/USP22/SIRT1/SOST, our results are still to be further verified. Above all, p38 MAPK mediates the expression of USP22 and SIRT1/SOST to suppress the occurrence of RB (**Figure 8**). This study expounds a potential molecular mechanism of p38 MAPK in the therapy of RB. In the future, more scrupulous and logical studies are required to support a promising clinical application in treatment for RB. 

## Data Availability Statement

The original contributions presented in the study are included in the article/**Supplementary Material**. Further inquiries can be directed to the corresponding authors.

## Ethics Statement

The studies involving human participants were reviewed and approved by Ethics Committee of Tianjin Medical University Eye Hospital. Written informed consent to participate in this study was provided by the participants’ legal guardian/next of kin. The animal study was reviewed and approved by Ethics Committee of Tianjin Medical University Eye Hospital.

## Author Contributions

XH and JW designed the study. XH, YL, FL, LW, and SZ collated the data, carried out data analyses and produced the initial draft of the manuscript. TW and FS contributed to drafting the manuscript. All authors contributed to the article and approved the submitted version.

## Funding

This work was supported by Tianjin Municipal Education Commission Scientific Research Project (Natural Science) NO.2020KJ176. Autonomous and open project of Tianjin Key Laboratory of Retinal Funtions and Diseases (NO.2020tjswmq002). Tianjin Municipal Key Clinical Discipline (Specialty) Construction Project (NO.TJLCZDXKQ018). Autonomous and open project of Tianjin Key Laboratory of Retinal Funtions and Diseases (NO.2019tjswmq001).

## Conflict of Interest

The authors declare that the research was conducted in the absence of any commercial or financial relationships that could be construed as a potential conflict of interest.

The reviewer BS declared a shared affiliation with several of the authors, XH, JW, SZ, TW, and FS to the handling editor at time of review.

## Publisher’s Note

All claims expressed in this article are solely those of the authors and do not necessarily represent those of their affiliated organizations, or those of the publisher, the editors and the reviewers. Any product that may be evaluated in this article, or claim that may be made by its manufacturer, is not guaranteed or endorsed by the publisher.
