# Supplementary material for: P38 Mitogen-Activated Protein Kinase Protects Against Retinoblastoma Through Regulating USP22/SIRT1/SOST Axis
Source: Front Oncol. 2022 Mar 9;12:781247. doi: 10.3389/fonc.2022.781247 (PMC8959650; doi:10.3389/fonc.2022.781247)
Supplement: Supplementary file 1 [file Table_1.docx]

**Primer sequences**

| Gene | Primer sequence |
| --- | --- |
| p38 MAPK | F: CGAAATGACCGGCTACGTGG |
|  | R: CACTTCATCGTAGGTCAGGC |
| USP22 | F: CTCCTGTCTGGTCTGTGAGATG |
|  | R: CAGCAACTTATACGGGATGTGA |
| SIRT1 | F: CATAGACACGCTGGAACAGG |
|  | R: GCAGATGAGGCAAAGGTT |
| SOST | F: CCGGAGCTGGAGAACAACAAG |
|  | R: GCACTGGCCGGAGCACACC |
| GAPDH | F: GACAAGATGGTGAAGGTCGG |
|  | R: CATGGACTGTGGTCATGAGC |
